# Dietary diversity and associated factors among pregnant women in Ethiopia: a systematic review with meta-analysis

**DOI:** 10.7189/jogh.15.04286

**Published:** 2025-10-24

**Authors:** Zenebu Begna Bayissa, Tsinuel Nigatu Girma, Jerusalem Azene Alamirew, Robera Olana Fite, Kassahun Alemu, Lisanu Taddesse, Delayehu Bekele, Getachew Tolera, Grace J Chan, Stefania I Papatheodorou, Bizu Gelaye

**Affiliations:** 1 Health System and Reproductive Health Research Directorate, Ethiopian Public Health Institute, Addis Ababa, Ethiopia; 2 Department of Public Health, College of Medicine and Health Sciences, Ambo University, Ambo, Ethiopia; 3 School of Population and Public Health, University of British Columbia, Canada; 4 Health and Health-Related Regulatory Directorate, Amhara Regional Health Bureau, Bahir Dar, Ethiopia; 5 HaSET Maternal and Child Health Research Program, Addis Ababa, Ethiopia; 6 Department of Obstetrics and Gynecology, Saint Paul's Hospital Millennium Medical College, Addis Ababa, Ethiopia; 7 Deputy Director General Office for Research and Technology Transfer Directorate, Ethiopian Public Health Institute, Addis Ababa, Ethiopia; 8 Department of Pediatrics, Children’s Hospital of Philadelphia, Perelman School of Medicine, University of Pennsylvania, Philadelphia, Pennsylvania, USA; 9 Department of Epidemiology, Harvard T.H. Chan School of Public Health, Boston, Massachusetts, USA; 10 Epidemiology Branch, Division of Population Health Research, Division of Intramural Research, Eunice Kennedy Shriver National Institute of Child Health and Human Development, Bethesda, Maryland, USA

## Abstract

**Background:**

Dietary diversity (DD) is the consumption of a variety of different and healthy foods that promote an adequate supply of nutrients, a high-quality diet, and the maintenance of optimal health. It is critical to identify the factors that influence pregnant women's eating habits so that relevant interventions can be developed. We estimated the pooled odds ratio of appropriate dietary practices to identify factors that affect the dietary practices of pregnant women.

**Methods:**

We conducted an electronic-based systematic search for observational studies conducted in Ethiopia and published in English. We retrieved published articles up to the last search date on 1 July 2022, from databases, including the Web of Science, PubMed, EMBASE, Cochrane library, CINHAL, and HINARI. Grey literature was included from Google and Google scholar searches. The measure of effect was a pooled odds ratio examining the association between the risk factors and adequate dietary diversity among pregnant women. The Cochran’s-Q statistic and *I*^2^ statistic tests with corresponding *P*-values were used to determine the existence of heterogeneity between studies. Publication bias was tested using a funnel plot of symmetry and further investigated using Egger and Begg tests. The results were presented using forest plots, funnel plots, tables, and figures.

**Results:**

We included 29 articles with maximum and minimum sample sizes of 759 and 241, respectively. Among the included articles, 13 were facility-based cross-sectional studies;16 studies were community-based cross-sectional studies. The pooled proportion of adequate DD was 42.48% (95% confidence interval (CI) = 31.82, 53.14). Knowledge of DD (OR = 3.10; 95% CI = 1.92, 4.99), income (OR = 0.35; 95% CI = 0.14, 0.85), and nutritional information (OR = 1.91; 95% CI = 1.15, 3.17) were predictors for adequate DD practice among pregnant women.

**Conclusions:**

The pooled proportion of adequate DD among pregnant women was low. Knowledge of DD, household income, and nutritional information were associated factors with the adequate dietary diversity of pregnant women. We recommend focusing on interventions that will enhance the knowledge of dietary diversity through improved nutritional awareness and enhance access to food resources through existing maternal health initiatives.

**Registration:**

PROSPERO: CRD42022298172

Pregnancy is a critical period during which major physiologic and metabolic changes occur [1]. During pregnancy, there is an increased requirement for micronutrients, energy, and macronutrients to support the mother's health and foetal development [2–4]. In sub-Saharan Africa, maternal undernutrition remains a public health concern. This includes Ethiopia, where the prevalence and incidence of maternal morbidity and mortality, low birth weight, preterm delivery, and childhood stunting are high [5]. Macro- and micronutrient deficiencies have been identified as the primary causes of these conditions, which result from poor access to nutrient-dense foods, such as animal products, fruits, vegetables, and fortified foods [6,7].

Dietary diversity (DD) is the consumption of a variety of different and healthy foods that promote an adequate supply of nutrients, a high-quality diet, and the maintenance of optimal health. It is one of the best strategies to positively impact maternal and foetal health [8,9]. There is a lack of consistent evidence in Ethiopia regarding pregnant mothers' dietary practices. Identifying the DD throughout the calendar year could contribute to planning and implementing maternal and child health programmes focused on nutrition. The results could support policymakers in making evidence-informed decisions on improving the nutritional outcomes of mothers and children. They could also contribute to the broader scientific community by identifying additional areas of research focused on pregnant women's dietary practices. We estimated the pooled proportion of DD of pregnant women in Ethiopia and the factors affecting their dietary practices.

## METHODS

### Design and setting

This review protocol is registered in the International Prospective Register of Systematic Reviews (PROSPERO) with the registration number (CRD42022298172). The results of the review followed the 2020 Preferred Reporting Items for Systematic Reviews and Meta-Analyses (PRISMA) (Table S1 in the **Online Supplementary Document**) guidelines and the Cochrane Handbook for Systematic Reviews Version 6.2 (John Wiley & Sons (Wiley-Blackwell)Ltd, London, UK 2021) [10,11].

Study selection included research that measured a pregnant woman’s food consumption using a qualitative survey based on dietary recall of nine or ten food items eaten over 24 hours. Dietary diversity was considered adequate if the individual consumed four or more of the nine food items and five or more of the ten food items [6,7,12]. The exposure variables of interest were sociodemographic and economic factors, obstetrics and pregnancy-related variables, and variables related to dietary practices.

### Eligibility criteria

In our review, we included studies using a combination of condition, context, and population (CoCoPop) criteria and those using an observational design such as descriptive, cross-sectional, case-control, and cohort studies. We included studies that reported dietary practice as an outcome variable for the condition and, to indicate the context, we included studies conducted in Ethiopia. All studies that enrolled pregnant women were included. We included all English articles published up to the last search date on 1 July 2020. All studies were included regardless of publication status. We excluded articles whose full text could not be obtained online or after three failed attempts to communicate through email with the corresponding authors. Furthermore, conference abstracts and journal editorials were excluded.

### Information sources

Web of Science, Scopus, PubMed/Medline, HINARI, EMBASE, CINAHL, and the Cochrane Library databases used retrieve published articles. Google and Google Scholar used for grey literature search. We combined search terms including prevalence, magnitude, incidence, risk factors, associated factors, determinants, predictors of dietary practice, dietary habits, nutritional status, nutritional habits, nutritional practices, dietary diversity, pregnant, pregnancy, and pregnant women. We also included Medical Subject Headings (MeSH) terms to search PubMed.

### Data screening

Initially, two independent reviewers ZBB and JAA screened the titles and abstracts and removed studies that did not fit the pre-defined screening criteria and the review objectives. The reviewers independently reviewed the full-text articles of the selected studies using full-eligibility criteria. Any discrepancies between the two reviewers were fixed through discussion and a third reviewer BG. We used EndNote V 7 to import references from the databases searched to avoid duplication.

### Data extraction and management

A detailed data extraction checklist, based on study characteristics and variables of interest, was developed and piloted to guide the systematic review process. The data extraction tool included information on the author’s name, title, data collection period, publication year, study population, study design, study setting, geographic region, sample size, response rate, and the raw data for adequate DD and inadequate DD. To identify the factors associated with adequate DD, we extracted raw data for each category of the outcome and exposure variables using Microsoft Excel. Then, the extracted data were compared, and disagreements were resolved by discussions between two reviewers and senior authors TG and BG.

### Outcomes and prioritisation

The primary outcome was the proportion of pregnant women with adequate DD, and the secondary outcome was the pooled risk factors of adequate DD.

### Quality appraisal

An adapted version of the Newcastle Ottawa Scale (NOS) of quality assessment used to appraise the quality of included studies. The quality assessment was done independently by ZBB and JAA. The NOS contained three major criteria: selection, comparability, and outcome. The selection criteria included issues such as representativeness of the sample, sample size, non-respondent, and ascertainment of the exposure (risk factors). The comparability criteria evaluated the assessment of the study participants in different outcome groups and if the confounding factors were controlled or not. The third criteria focused on how the outcome was assessed and the use of appropriate statistical tests. Studies with NOS scores of six or higher were considered as ‘good’ quality, while studies with NOS scores of six or less were considered as ‘poor’ quality [13]. The Grading of Recommendations Assessment, Development, and Evaluation (GRADE) was employed to evaluate the quality of evidence for all outcome categories. The quality of evidence was assessed across five domains, including risk of bias, consistency, directness, precision, and publication bias.

### Data analysis and synthesis

Extracted data was entered into Microsoft Excel and then exported to Stata version 17, (StataCorp LLC, Texas, USA) software for further analysis. A narrative synthesis was done on the studies included, the study population, and risk factors of DD practices. The pooled odds ratio with 95% confidence intervals of the adequate DD of pregnant women was computed using the random-effects analysis model [14,15]. A leave-one-out sensitivity analysis was performed to evaluate the main studies with the greatest impact on between-study heterogeneity. The analysis was performed to assess each study's effect on the pooled estimated prevalence by excluding one study at a time. The results were presented using forest plots, funnel plots, tables, and figures.

### Heterogeneity and publication bias

The existence of heterogeneity between studies were checked using Cochran’s-Q and *I*^2^ statistic tests. In this review, a value of *I*^2^ of 25% was considered low, 50% moderate, and 75% high [16]. When the results showed the presence of between-study heterogeneity, we conducted subgroup analyses, meta-regression, and sensitivity analysis to explore heterogeneity and to observe changes over a year. Publication bias was tested using a funnel plot of symmetry and further investigated using Egger and Begg tests. A *P*-value of <0.05 considered as publication bias [17,18].

## RESULTS

### Selection and identification of studies

We found 3935 (3932 published and three unpublished) articles from our search. Only 29 articles were eligible for the review and meta-analysis (Figure S1 in the **Online Supplementary Document**). Of these, 13 articles were based on facility-based cross-sectional studies, and 16 were from community-based cross-sectional studies. The maximum sample size included was 759, and the minimum was 241.

Six Ethiopian regions (South Nation Nationality People (SNNP), Oromia, Amhara, Afar, Sidama and Tigray) and two city administration (Addis Ababa and Dire Dawa) were represented. Eight studies were in the SNNP; one from Sidama region, eight were in the Oromia region, eight were in the Amhara region, one each in Afar, Addis Ababa, Dire Dawa city, and Tigray. The maximum proportion of adequate dietary practice, (96.65%), and the minimum (7.9%) were observed in the studies conducted in Oromia and SNNP regions, respectively (Figure S1 in the **Online Supplementary Document**).

### Quality assessment of included studies

According to the NOS, all included studies were of good quality: 15 (51.7%) had a score of seven [6,14,19–31] had a score of eight [13,32–49].

### The proportion of dietary diversity among pregnant women

**Figure 1:**
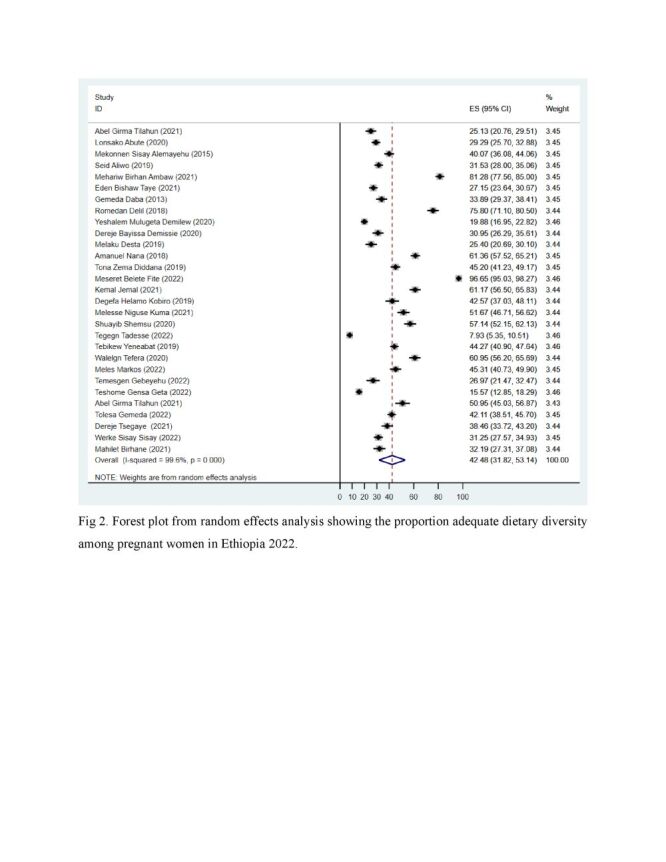
Proportion of adequate dietary diversity among pregnant women, Ethiopia, 2022.

We observed that the proportion of adequate DD among pregnant women varied between 7.93% [28] and 96.65% [36] across the studies. The *I*^2^ consistency and Cochrane Q heterogeneity test statistics showed high heterogeneity (*I*^2^ = 99.6, *P* < 0.0001) (**Figure 1**). The pooled proportion of DD was 42.48% (95% CI = 31.82, 53.14) (**Figure 1**).

### Subgroup analysis

We conducted subgroup analyses by the study regions, study types, and publication year to identify the potential source of heterogeneity, but found none (Figure S2–4 in the **Online Supplementary Document**). The review had no publication bias (Figure S5 in the **Online Supplementary Document**), and no strong evidence for the influence of a single study (Figure S6 in the **Online Supplementary Document**).

### Factors associated with dietary diversity of pregnant women.

We assessed the association of knowledge of dietary diversity, maternal education, maternal occupation, household income, nutritional information, household food security, and place of residence with adequate DD of pregnant women.

### Knowledge about dietary diversity

**Figure 2:**
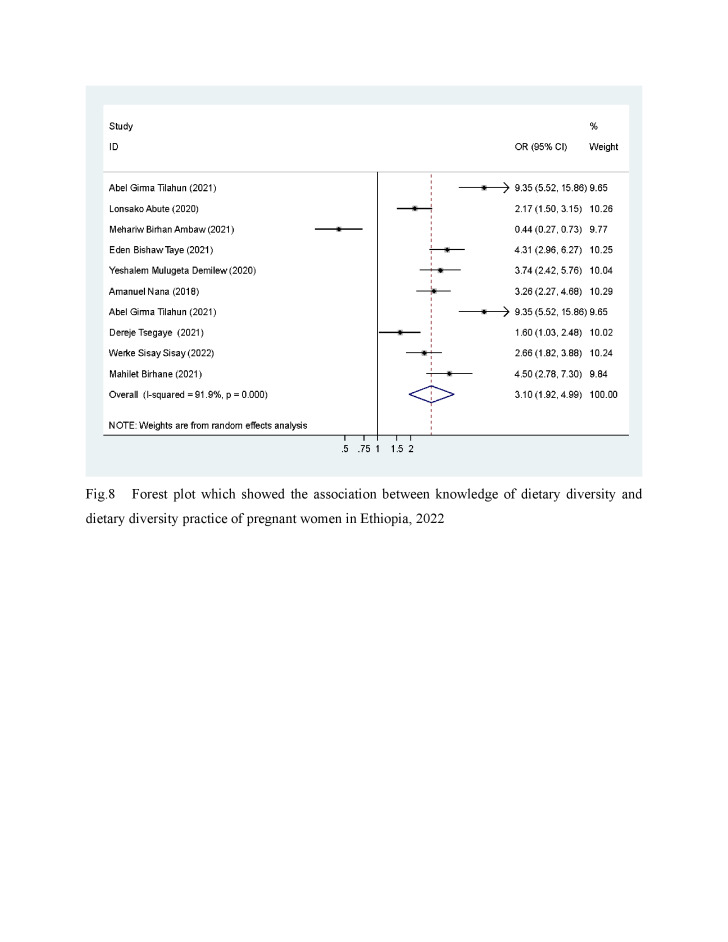
Association between knowledge of dietary diversity and dietary diversity practice of pregnant women: a systematic review and meta-analysis, Ethiopia, 2022.

Ten studies [6,20,23,25,31,32,34,42,45] assessed the association of knowledge with the dietary diversity of pregnant women. Women with good knowledge of dietary diversity had higher odds of adequate DD than women with poor knowledge about DD (OR = 3.10; 95% CI = 1.92, 4.99) (**Figure 2**). This indicates that individuals with knowledge about dietary diversity had almost three times higher odds of consuming a dietary diversity.

### Maternal education

We assessed 18 [6,8,19,20,28–31,33,34,36,39–41,44,45] articles to observe the association between maternal education and the practice of DD among pregnant women. Accordingly, the pooled OR was 1.69 (95% CI = 0.83, 3.44), which is not statistically significant (Figure S7 in the **Online Supplementary Document**).

A sensitivity analysis was done to rule out the influence of a single study with the highest effect size in the overall meta-analysis [20,44]. There was no influence of a single study (Figure S8 in the **Online Supplementary Document**), and no publication bias.

### Income

**Figure 3:**
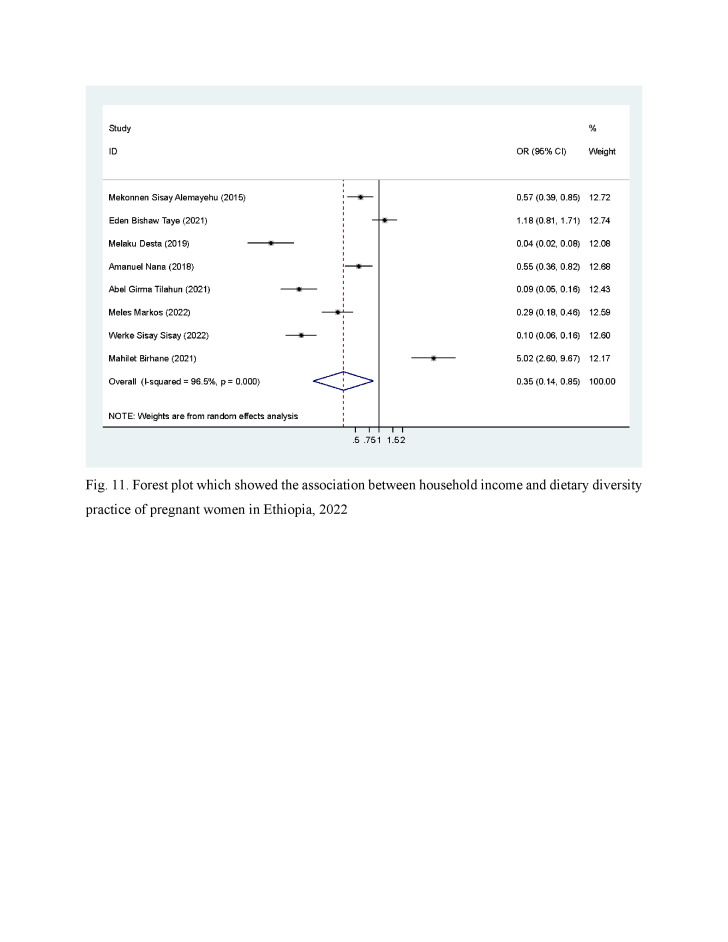
Association between household income and dietary diversity practice of pregnant women: systematic review and meta-analysis, Ethiopia, 2022.

We assessed eight articles to observe the association of income with the DD of pregnant women. Accordingly, pregnant women with a household income greater than two thousand Ethiopian birr have better probability of adequate DD practice than who have less than 36.34 USD (2000 Ethiopian birr) per month (**Figure 3**)

### Information about nutrition

**Figure 4:**
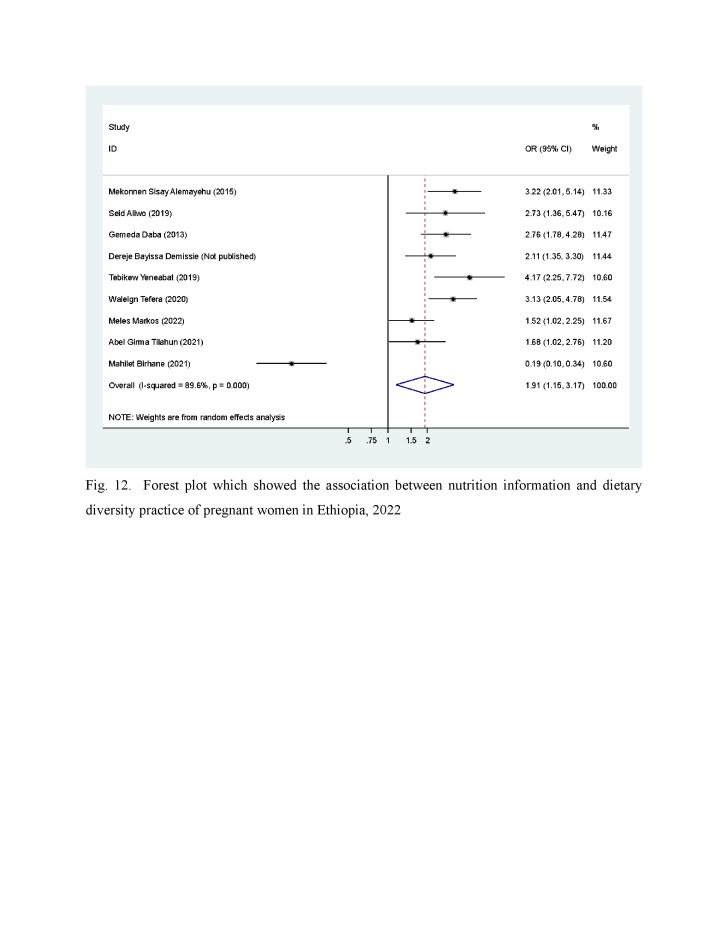
Association between nutrition information and dietary diversity practice of pregnant women: systematic review and meta-analysis, Ethiopia, 2022.

Nine [8,21,24,29,33,40,41,43,45] articles were included to assess the association of nutrition information with dietary diversity practice among pregnant women. Our analysis indicated that the odds of having adequate DD practice among women who received nutrition information was OR = 1.91 (95% CI = 1.15, 3.17) compared with those who did not receive nutritional information (**Figure 4**). This indicates that individuals with access to information had almost two times higher odds of consuming a dietary diversity.

### Food security

In seven studies [23,26,31,32,37,44] the researcher observed the association between food security with DD practices of pregnant women. The current meta-analysis revealed a statistically insignificant association of OR of 1.37 (95% CI = 0.54, 3.48) between food security and DD practice (Figure S9 in the **Online Supplementary Document**).

### Residence

Six studies [22,33,36,39,42,44] were examined to assess the association between residence and DD practice. The result of our analysis was not statistically significant, *P*-value = 0.98 (95% CI = 0.22, 4.25). We conducted a bias analysis to identify the effect of a single study, and there was a statistically significant result for the Egger and Beg test (*P*-value:0.29 (95% CI = −92.64, -36.71) and 1.00), respectively (Figure S10).

### Occupation

Nine studies [6,8,21,27,30,36,37,42,43] were examined to observe the association between maternal occupation and DD practices of pregnant women. We did not find the association statistically significant (*P*-value = 0.61 (95% CI = 0.29, 1.28)) (Figure S11 in the **Online Supplementary Document**).

## DISCUSSION

The current systematic review and meta-analysis, reported that the pooled proportion of DD was 42.48%. Dietary Diversity practices of pregnant women were significantly associated with income, nutrition information, and knowledge of dietary diversity, but not maternal education, food security, occupation, and residence.

The pooled proportion of adequate dietary diversity in our study varied from two previous Ethiopian studies: in 2020 the pooled prevalence of inadequate DD was 53%, and in 2021, the pooled prevalence of medium DD was 41.55% in pregnant women and 41.22% in lactating women [46,47]. Our finding of 42.48% could be due to the continuous implementation of strategies and activities at the national level to improve maternal and child health.

The odds of adequate DD among pregnant women were significantly higher among women with a monthly income of greater than 36.34 USD (2000 Ethiopian birr). It is well established that improving income can significantly improve the ability to purchase food and increase access to health care services [13]. This finding is supported by studies conducted in many LMICs including in Senegal, Nigeria, Iran, and India, and indicates how an inadequate economic environment can lead to malnutrition [13,48]. Improving family income and enhancing access to food resources has direct implications for DD, especially in a LMIC country such as Ethiopia [48].

In addition, this analysis showed that DD was significantly associated with the knowledge of pregnant women about DD. Our review identified hat the odds of having adequate DD was 3.10 (1.92, 4.99) among women who had better knowledge about DD than poor knowledge. In our analysis, we also observed that having nutritional information was positively associated with DD. When women understand the benefits of eating a diverse diet during pregnancy for the babies’ health, and know where to access the essential nutritional information, they may be able to make changes to their dietary practices [50].

We found that food security, residence, and maternal occupation were not statistically significant in the DD practice of pregnant women. For all three variables, a sensitivity analysis was done to identify the influence of a single study, but none was identified. This could be due to the small number of studies included, or the difference in the sample size among the studies included. We therefore recommend conducting a qualitative study for these variables to understand the link between them and DD practice among pregnant women.

The current review showed the presence of heterogeneity. Although we conducted subgroup analysis by study design, region, and time of publication, the source of heterogeneity was not due to these variables. We also conducted a sensitivity analysis but could not identify the effect of a single study. Therefore, the source of heterogeneity could be due to uncontrolled bias in the original studies or might be due to other factors which were not considered by the present review.

One of the limitations of this review was that all the studies included were cross-sectional in design and did not provide any evidence of temporality between the factors and DD. Prospectively designed studies are needed to understand the causal relationship between risk factors and maternal DD. In addition, the included studies may not have represented all regions of the country. Finally, the COVID-19 pandemic created an unprecedented impact on food security that this study could not capture.

## CONCLUSIONS

The pooled prevalence of DD in Ethiopia is low compared to the global recommendation. Based on our review findings, income, nutritional information, and knowledge about DD were significantly associated with the DD practice of pregnant women. Thus, the focus should be on interventions that will enhance the knowledge of DD through improved nutritional awareness and enhance access to food resources through existing maternal health initiatives. Moreover, qualitative studies are recommended to understand the link between maternal education, occupational, and food security with DD from the lived experience of pregnant women.

## Additional material


Online Supplementary Document
