# Supplementary material for: Dietary diversity and associated factors among pregnant women in Ethiopia: a systematic review with meta-analysis
Source: J Glob Health. 2025 Oct 24;15:04286. doi: 10.7189/jogh.15.04286 (PMC12550538; doi:10.7189/jogh.15.04286)
Supplement: Online Supplementary Document [file jogh-15-04286-s001.pdf]

Supplement to: : Bayissa ZB, Nigatu TG, Azene JA, Fite RO, Alemu K, Taddesse L, Bekele D, Chan GJ, Gelaye B. Dietary diversity and associated factors among pregnant women in Ethiopia: a systematic review with meta-analysis. J Glob Health. 2025;15:04286.

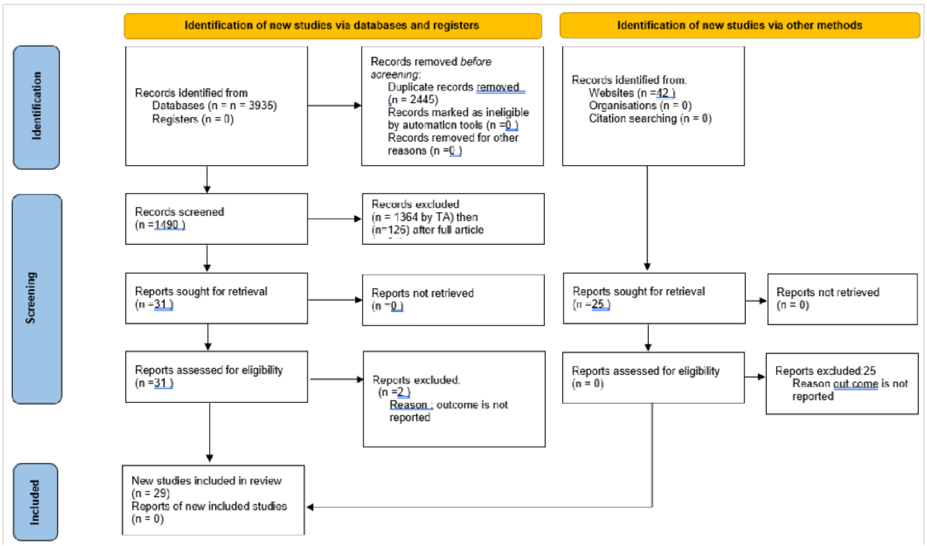

Fig 1. PRISMA diagram of studies included in the systematic review and meta-analysis.

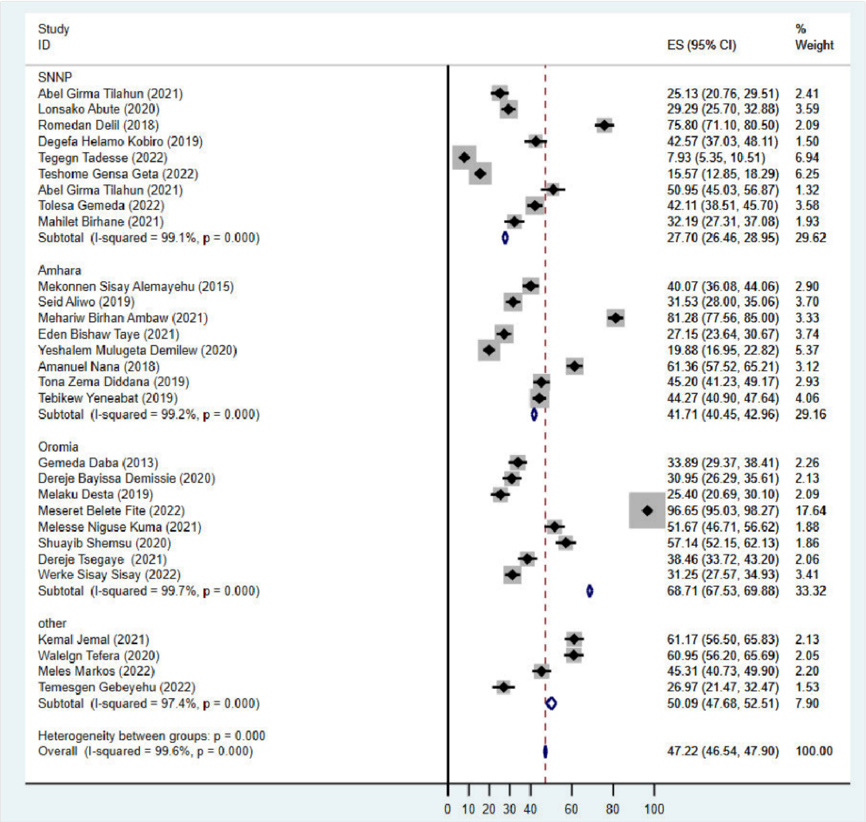

Figure 2. Forest plot of proportion of dietary diversity by region of residence among pregnant women in Ethiopia, 2022.

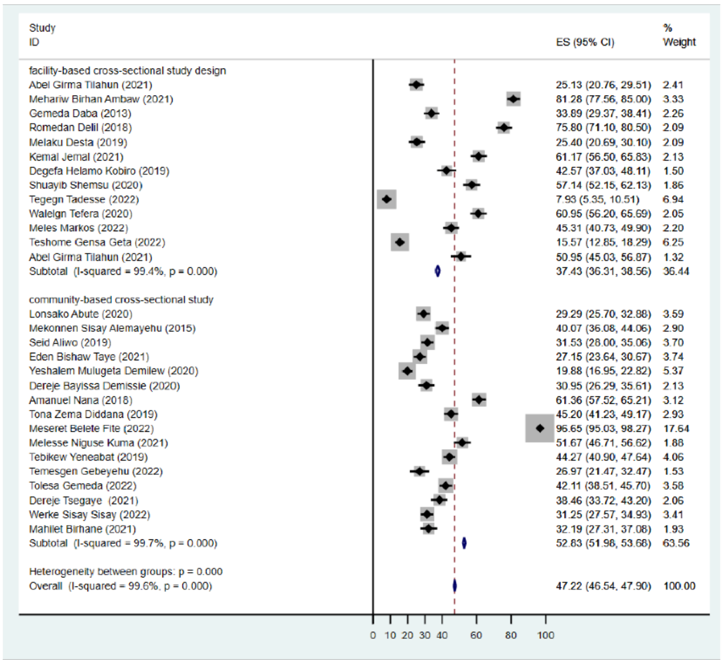

Figure 3. Forest plot of proportion of adequate dietary diversity by study type among pregnant women in Ethiopia, 2022.

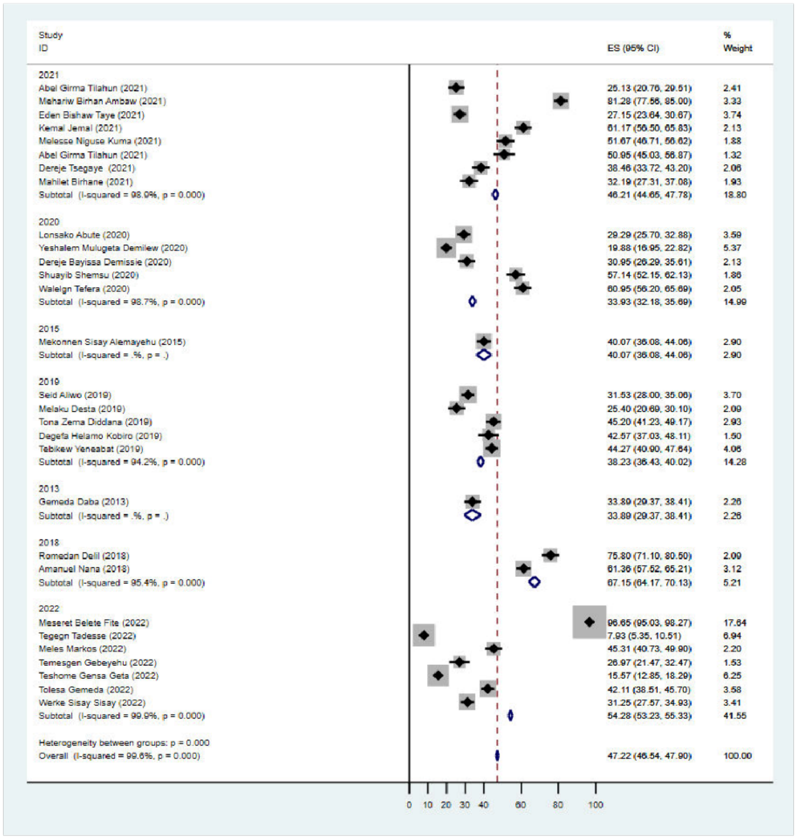

Figure 4. Forest plot of proportion of dietary diversity by study publicaton year among pregnan women in Ethiopia, 2022.

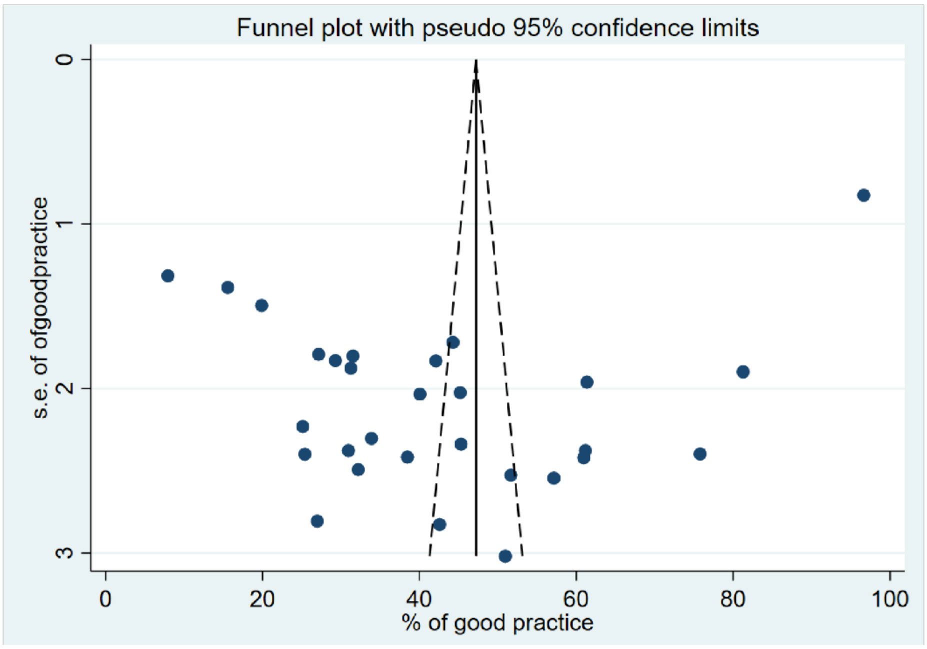

Figure 5. Funnel plot for publication bias for the proportion of adequate dietary diversity among pregnant women i Ethiopia 2022.

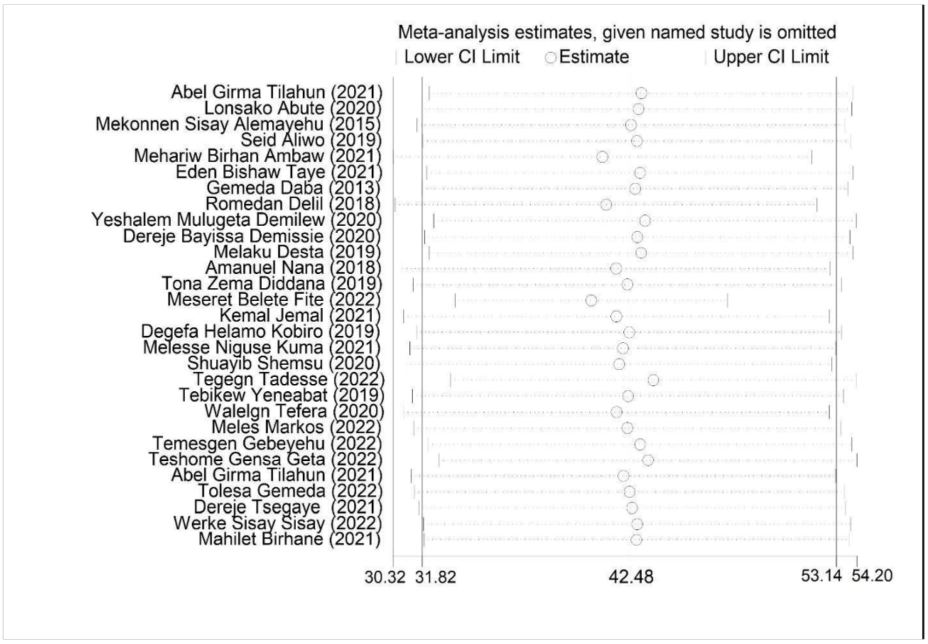

Figure 6. Result on the effect of a single study on the meta-analysis estimates of the dietary practice of pregnant women in Ethiopia.

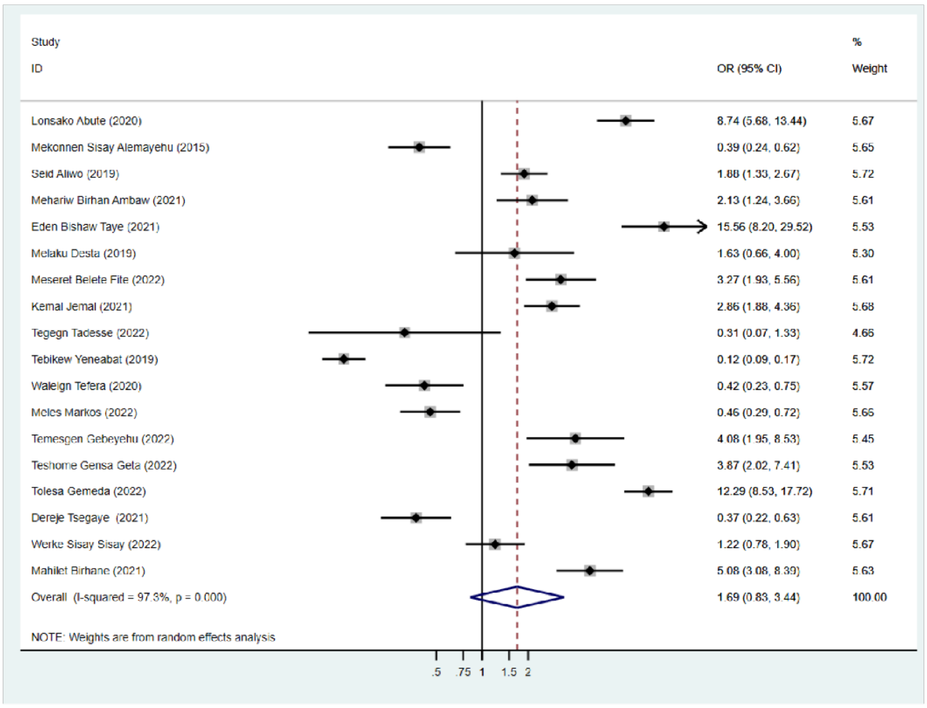

Figure 7. Forest plot which showed the association between maternal education and dietary diversity practice of pregnant women Ethiopia, 2022.

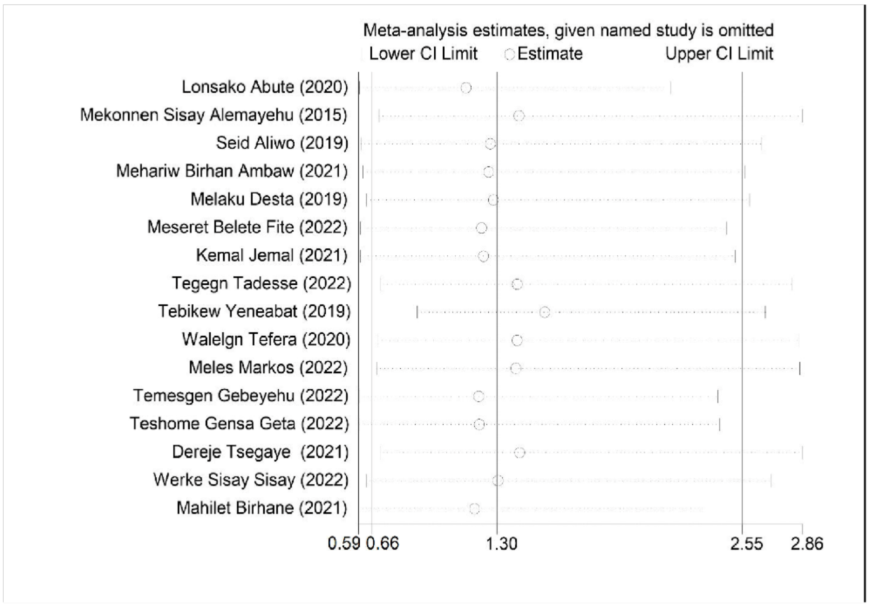

Figure 8. A sensitivity analysis for maternal education to analyze the effect of a single study on the meta-analysis estimates of the dietary practice of pregnant women.

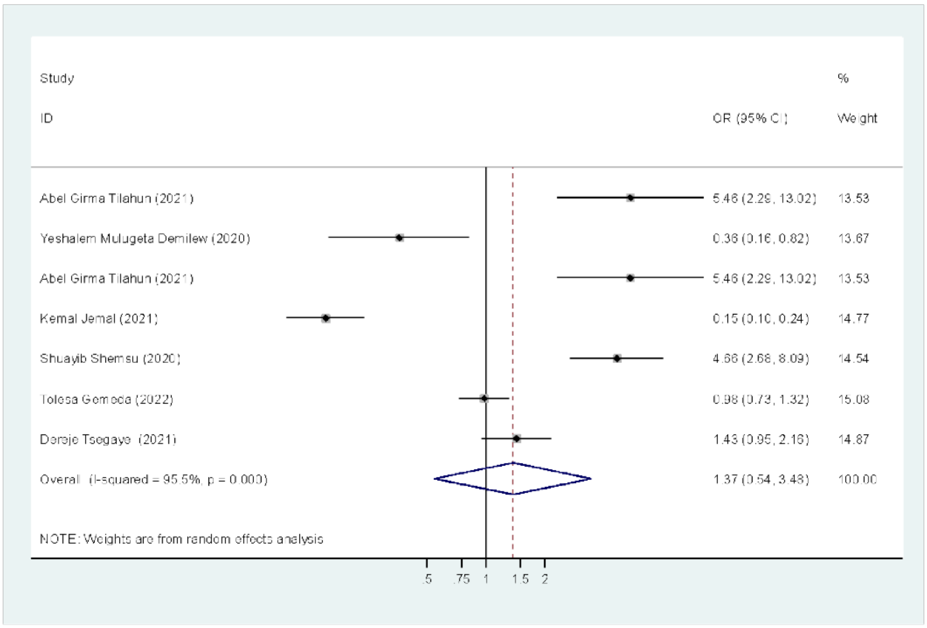

Figure 9. Forest plot which showed the association between food security and dietary diversity practice of pregnant women in Ethiopia, 2022.

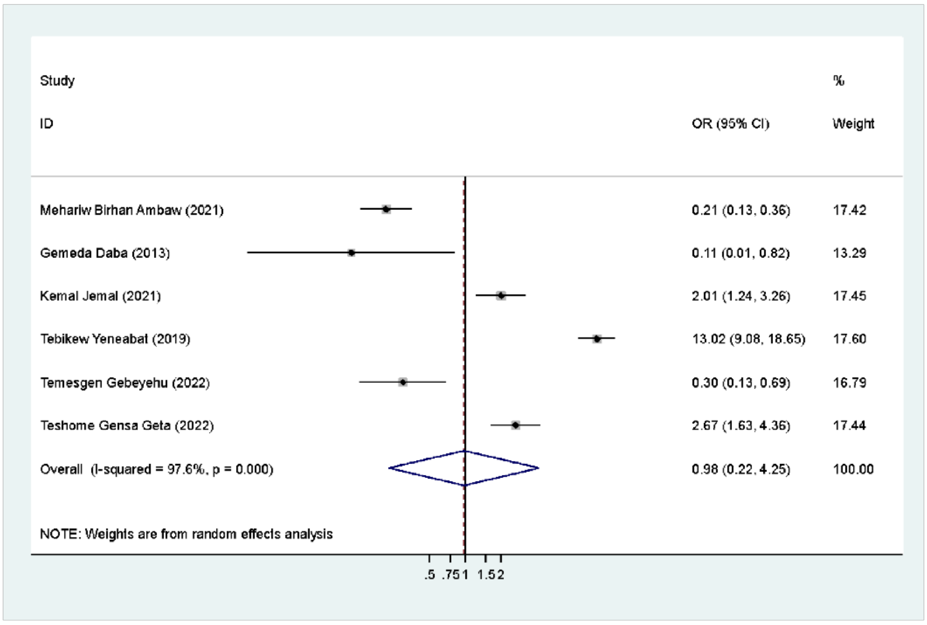

Figure 10. Forest plot which showed the association between residence and dietary diversity practice of pregnant women in Ethiopia, 2022.

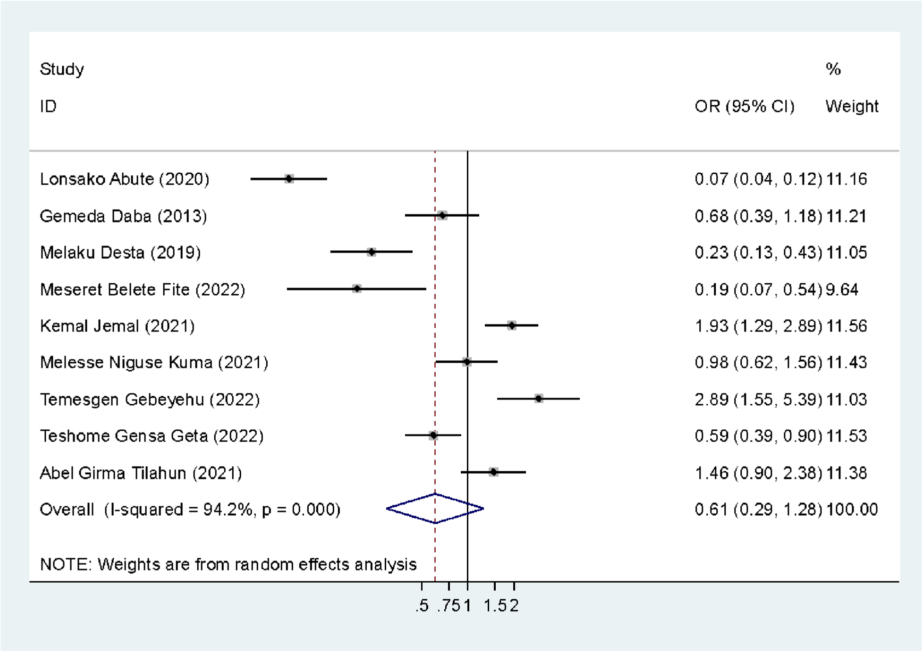

Figure 11. Forest plot which showed the association between occupation and dietary diversity practice of pregnant women in Ethiopia, 2022.
